# The talent study: a multicentre randomized controlled trial assessing the impact of a ‘tailored lifestyle self-management intervention’ (talent) on weight reduction

**DOI:** 10.1186/s40608-015-0069-x

**Published:** 2015-10-01

**Authors:** Dieter Melchart, Wolfgang Doerfler, Axel Eustachi, Yanqing Wellenhofer-Li, Wolfgang Weidenhammer

**Affiliations:** Competence Centre for Complementary Medicine and Naturopathy, Klinikum rechts der Isar, Technische Universität München, Munich, Germany; Institute for Complementary and Integrative Medicine, University Hospital Zurich and University of Zurich, Zurich, Switzerland

**Keywords:** Individual Health Management (IHM), Overweight, Prevention, E-lifestyle modification, Diet, RCT, Network of health promotion, Web-based health portal

## Abstract

**Background:**

Overweight is considered an important risk factor for diseases in the context of metabolic syndrome. Lifestyle modifications are the means of choice to reduce weight in persons with a Body Mass Index of 28 to 35. The study examines whether there are any differences between two intervention strategies regarding weight reduction in overweight persons.

**Methods/Design:**

The study is a multicentre randomized controlled trial with observation duration of 12 months. Eight study centres are involved to include a minimal sample size of 150 participants. Randomization ratio is 2:1. Feasible persons are checked according to inclusion and exclusion criteria and after given informed consent are assigned randomly to one of two intervention programs: A) intervention group: comprehensive lifestyle modification program (Individual Health Management IHM) with 3 months reduction phase plus 9 months maintaining phase, B) control group: written information with advice for healthy food habits (Usual care UC). Participants of the IHM group have access to a web-based health portal and join 3 full-day and 10 two-hour training sessions during the first 3 months. During the remaining 9 months four refresh trainings will be performed. There are 3 different diet strategies (fasting, two-day diet, meal replacement) for free choice. Participants of the control group are provided with acknowledged rules for healthy food according to the German Nutrition Society (DGE). Examinations are conducted at baseline, after 3, 6, 9 and 12 months. They include body weight, waist circumference, blood pressure, laboratory findings and a bio-impedance analysis to measure body composition. Statistical analysis of the primary outcome ‘change of body weight after 12 months’ is based on ITT population including analysis of variance of the weight differences between month 0 and 12 with the factors ‘group’, ‘baseline value’ and ‘study centre’. Secondary outcomes will be analyzed exploratively.

**Discussion:**

The monitoring of the study will implement different measures to enhance compliance, avoid attrition and ensure data quality. Based on a blended learning concept and using web-based e-health tools the program promises to achieve sustainable effects in weight reduction.

**Trial registration:**

German Clinical Trials Register Freiburg (DRKS): DRKS00006736 (date registered 20/09/2014).

**Electronic supplementary material:**

The online version of this article (doi:10.1186/s40608-015-0069-x) contains supplementary material, which is available to authorized users.

## Background

There is an increasing trend for overweight and obesity worldwide. In 2008, 35 % of adults aged 20+ were overweight (BMI ≥ 25 kg/m^2^). The worldwide prevalence of obesity has nearly doubled between 1980 and 2008 [1]. In a more recent analysis this trend has been confirmed [2]. In developed countries like US the figures are markedly higher. Approximately two thirds of adults are overweight (BMI of 25-29.9) or obese (BMI ≥ 30) [3]. The German Health Interview and Examination Survey for Adults (DEGS1), conducted from 2008 through 2011, provides current data about overweight and obesity among adults in Germany based on a representative sample of 7116 persons [4]. Prevalence of overweight defined as BMI ≥ 25 is 53 % for females and 67 % for males with increasing rates in higher age groups. While prevalence of obesity grade I (BMI 30-35) is similar in both sexes (16 % in women, 18 % in men) pre-adipositas (BMI 25–30) is more frequent in men (44 %) than in women (29 %).

Obesity is associated with many comorbid conditions which has major implications for longevity, quality of life and healthcare costs [5]. It could be shown that a strong and linear association exists between Body-Mass-Index (BMI > 20) and the risk of developing type 2 diabetes, hypertension, CVD, and other chronic diseases in both men and women [6].

Therapeutic interventions aiming at weight reduction range from multiple modalities including behavioural therapy and pharmacotherapy integrating clinical and community approaches [7] to lifestyle counselling and mere nutritional programs. Enhanced lifestyle counselling proved to be superior to usual care with respect to weight reduction [8]. Compared to minimal or no intervention it could be shown that self-management plus exercise prevented weight increase in patients with cardiometabolic disease [9]. A systematic review comparing the effects of different popular diets revealed that only weight-watchers program was beneficial to usual care in RCTs of 12 month duration [10].

From the findings in the literature one has to conclude that a sustainably successful treatment of overweight requires intensive education, counselling, multiple resources, and ongoing support. The Individual Health Management (IHM) is a program which meets these prerequisites. It is theory driven and designed to promote changes across multiple health behaviours simultaneously aiming for participation of healthy and ill people to enhance their individual responsibility, self-determination and health literacy [11]. Self-management supporting the participants to optimize physiological skills and psycho-social competencies is considered the core element of the intervention. It comprises areas like physical activity, nutrition, self-efficacy and social support. To enhance sustainability they are to be trained and implemented into daily activities. The lifestyle program combines a web-based lifestyle modification program, physician-led counselling via face-to-face or telemedicine visits and various forms of group sessions to achieve behavioural change (blended learning concept). To ensure a high standard of professional service, IHM health staff graduates in a 200 h theoretical and practical training in comprehensive life style medicine.

Although the basic concept of IHM is very broad and multifaceted the planned study will focus on weight reduction as target parameter. Due to a comprehensive change in lifestyle the effects are expected to be enduring. Thus, the decisive measurement of body weight should be one year after onset of the program. For weight monitoring in overweight persons including obesity grade I it is highly relevant to compare the effects of IHM with the common brief diet and lifestyle counselling used in daily routine of general practitioners. This is in line with the findings of a systematic review of RCTs on multifactorial lifestyle interventions in primary and secondary prevention [12].

The study is part of a comprehensive network program to install and maintain prevention programs for different risk groups at selected spas in Bavaria. The project is to contribute to the improvement of the medical quality of health services offered in the spas.

The primary objective of the study is to examine whether there are any differences between two intervention strategies (Individual Health Management versus usual care) regarding weight reduction at month 12 in overweight persons. Further objectives are the comparative analysis of secondary outcome parameters like Body-Mass-Index, waist circumference, laboratory findings, blood pressure and the ratio of fat, muscle mass and bodily water. Moreover, the descriptive comparison of both intervention groups include the analysis of the course of changes in body weight, a responder/non-responder analysis, and an analysis with respect to the applied dietary strategy.

## Methods/Design

### Study design

The study is a multicentre randomized controlled trial. The study duration for each participant is 12 months with five sequential examinations. It will be performed in total in 8 study centres located in different spa towns all over Bavaria, and complemented by the outpatient unit of the Competence Centre for Complementary Medicine and Naturopathy at Klinikum rechts der Isar, Munich. The centres in the spas are affiliated to private practices of General practitioners. These centres are committed to be a part of the local core teams promoting Individual Health Management (IHM) which are pooled into a centrally coordinated network of health promotion called ‘IHM-Campus’.

### Recruitment and participants

Measures for recruitment of feasible study participants include different strategies which are not regulated equally across all study centres. The methods consist of: search in the databases of the GP practices, conduct of local action days to attract population interested in overweight and prevention from diabetes, advertisements in local media with the offer for obese persons to participate in a clinical study. All interested persons will contact the local IHM teams which will provide basic information around the study and a tool to screen for the main inclusion criteria. All persons who apparently should comply with the requirements of the study are invited to a personal appointment with the trial physician to undergo a comprehensive examination of the criteria for inclusion and exclusion. Potential participants will be informed on background, objectives, benefit and risks of the study by a leaflet as well as orally by the trial physician.

### Inclusion/exclusion criteria

Persons of both sexes, aged 18 to 67 years, with moderate overweight defined as Body-Mass-Index from 28.0 to 35.0, can be included to the study. A written informed consent is mandatory.

Persons will not be included if one of the following exclusion criteria are present: not legally competent, insufficient skills in German language, no private access to internet, already known pregnancy, known psychiatric disease including eating disorder or addiction, known diseases of the eyes, known diabetes type 1 or 2, hypertension grade II (systolic BP ≥ 160 mmHg or diastolic BP ≥ 100 mmHg) with/without medication, known heart disease, known gastric or duodenal ulcers, diseases of the liver or kidneys which do not allow an increased intake of proteins, disease-related impairments hampering certain elements of the lifestyle program or therapeutic conditions not compatible with lifestyle modifications.

### Randomisation

Eligible participants will be randomized immediately after formal inclusion to the study. The trial physician will open the closed envelopes in strictly sequential order of the enrollments and the allocated treatment arm will be disclosed to the study participant. The allocation ratio is 2:1, means 2 for IHM group and 1 for the comparison group. Randomisation and allocation envelopes will be prepared by an independent data manager at the institute for medical statistics and epidemiology at TU Munich.

### Interventions

The study compares two intervention arms (IHM = Individual Health Management; UC = Usual Care):

- *Group IHM*: Lifestyle program ‘Individual Health Management’ with an overall duration of 12 months is composed of 2 phases: i) the first 3 months (reduction phase) include 3 full-day so called’introduction days’ plus 10 two-hour weekly training sessions; ii) during the following 9 months (maintaining phase) participants are practicing lifestyle modifications by oneself supported by 4 full-day refresh training sessions. The program encompasses access to a web-based health portal (https://www.viterio.de/) providing detailed advice and instructions with respect to food, exercise and relaxation. Furthermore, this tool allows a personalized feed-back control of the progress made so far. IHM is implemented as a group intervention program with a group size of roughly 12. All training sessions will be performed in the local study centres. Details of the intervention concept and realization are described elsewhere [11]. All participants will be offered three different diet strategies: i) fasting (per week one regular day, five waiver days, one food restriction day with < 900 kcal plus fasting during week 7), ii) two-day diet (five regular days, two serial food restriction days with < 600 kcal), iii) meal replacement (one regular day, five waiver days, one food restriction day with meal replacement < 900 kcal plus meal replacement in week 7) for free choice.
- *Group UC*: This intervention is an active control intervention reflecting the common practice in usual care. Participants are provided with a leaflet containing 10 acknowledged rules for healthy food and physical exercise according to the German Nutrition Society (DGE). They will neither join the group sessions nor will get access to the web-based health portal.

**Fig. 1:**
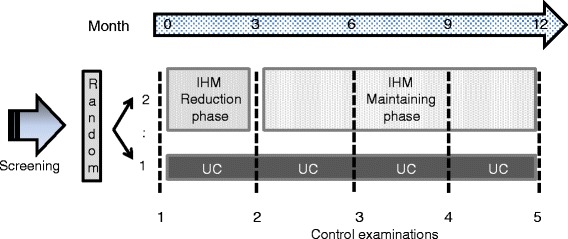
Flow chart showing the design of the study (IHM = Individual Health Management; UC = Usual Care)

Figure 1 shows the basic design of the study with the two treatment arms and all examinations.

### Outcomes

The primary outcome measure to test the hypothesis of no differences between the effects of both intervention groups is change in body weight from baseline to month 12. Secondary outcome parameters are Body-Mass-Index, waist circumference, blood pressure, heart frequency, laboratory findings, and distribution of bodily fat measured by bio-impedance-analysis. The occurrence of adverse events will be captured systematically at each physical examination following the baseline testing. Weight reduction will also be analyzed by the frequency of responders in both groups. A responder at month 12 will be defined as a 7.5 % reduction of baseline body weight. The schedule for all examinations during the study is displayed in Table 1.

**Table 1:** Study process chart

	Month				
Examination	0	3	6	9	12
Inclusion-/exclusion criteria^a^	X				
Informed consent^a^	X				
Randomisation	X				
Sociodemographic data	X				
Size	X				
Weight, waist circumference, blood pressure, heart frequency	X	X	X	X	X
Bio-Impedance-Analysis	X	X	X	X	X
Laboratory findings:					
Electrolytes (Ca, K, Cl, Mg, Na)	X				X
Liver enzymes (ASAT, ALAT, GGT, ALP, Bilirubin)	X				X
Kidney (Crea, Urea, Uric acid)	X				X
Blood count (Ery, Leuco, Ptl, Hk, Hb)	X				X
TSH (basal)	X				X
Fasten glucose	X	X			X
Trigycerides	X	X			X
Total cholesterol	X	X			X
HDL-/LDL-Cholesterol	X	X			X
Adverse events/effects		X	X	X	X

^a^to be checked before inclusion into study

### Statistical analysis

Based on indications from the literature we assumed a mean reduction for body weight after 12 months of 6 kg under IHM and of 3 kg under usual care conditions. Given a common standard deviation of 6 kg for the change in weight the sample size for the *t*-test for independent groups (α = 0.05, 2-sided, power 80 %) is estimated as 98 in one group and 49 in the second (allocation ratio 2:1). Hence, the total sample size is 150 participants.

The confirmatory analysis will be conducted using a 2-sided significance test at the 5 % significance level. Based on the intent-to-treat population the primary outcome (change in body weight Δ_month 0 – month 12_) will be tested by analysis of variance with the grouping factor ‘intervention’ controlled for baseline value. The factor ‘study centre’ will also be included to the test model. In participants with missing data for weight at month 12 (drop-outs) an adequate conservative imputation technique will be applied.

Secondary endpoints will be analyzed analogously but using an explanatory approach. Hence, a correction of the error probabilities due to multiple testing is not indicated. These analyses will be performed on both intent-to-treat and per-protocol populations. A responder analysis with respect to the primary outcome will be performed using a responder definition with a threshold of 7.5 % weight reduction at month 12 compared to the baseline value. Depending from the number of participants who chose the different nutritional regimen a subgroup analysis within the IHM group will be performed.

All captured data will be analyzed descriptively by appropriate statistical parameters: absolute and relative frequencies for categorical data and arithmetic means, medians, standard deviations for numerical data. Where indicated, 95 % confidence intervals will be presented.

Apart from the confirmatory analysis a series of sensitivity analyses will be performed to explore the impact of different factors on the results of the analyses. Relevant factors are among others adherence to the protocol, adherence to the intervention program IHM and different responder definitions.

### Ethical review

Ethical approval for this study was obtained from the ethical review board of the Medical faculty of TU Munich (file number 97/14). The investigators will ensure that the study will be conducted in compliance with the ethical guidelines as set out by this committee, and in line with the guidelines for good clinical practice (GCP).

## Discussion

### Quality assurance and bias

The protocol was developed according to the Consort guidelines (see Additional files 1 and 2). All trial physicians are qualified regarding the legal requirements according the German Drug Law (AMG) and in line with the guidelines for GCP and ICH. Staff members of the study centres at the participating spas being involved in coaching of the IHM group members are trained and certified as health coaches.

The study will be monitored by a professional institution with acknowledged expertise in supervision of clinical trials of all kinds (Munich Study Centre at the Medical faculty of TUM). The monitoring plan comprises a central indoor-monitoring as well as onsite visits at the participating study centres. The confirmatory analysis of the data is conceptualized and will be performed by an independent statistician at the institute for medical statistics and epidemiology at the TUM.

Although randomization should eliminate selection bias there are some other issues with potential impact on the conclusiveness of the study. First, we tried to avoid early drop-outs by comprehensive information of the study. Both intervention arms are introduced as strategies to reduce body weight, and the acceptance was enhanced by choosing a 2:1 allocation ratio assuming the IHM group as the more popular one. Participants allocated to the IHM group will have a free choice of different dietary strategies. So for example, if someone does not wish to do fasting, this is not to discourage him from participating in the study since there are other options for an individual dietary strategy.

Another issue is loss to follow-up which may lead to biased estimates of intervention effects. While during the first three months the high frequency of personal meetings in the IHM group warrants a close relationship between the study coaches and the participants there is more need for careful following up the members of the comparison group. We will remind them of the upcoming examination visits by phone or mail and consider the individual requirements to arrange feasible dates. We will implement procedures to minimize the number of drop-outs, and we will ask to tell us the reasons for withdrawal, where possible. Nevertheless we anticipate an attrition rate of about 10 %, and appropriate methods for handling these drop-outs in the statistical analysis will be applied (see ‘Statistical analysis’).

The potential effectiveness of an intervention is affected by non-adherence to the intervention program. The IHM program under investigation is requesting a high level of active participation. During the whole study there are sixteen personal meetings scheduled. Before enrollment to the study all potential study participants are informed in detail on the requirements. Although basically accepted it has to be anticipated that especially for those participants with a longer distance between their domicile and the study centre not all appointments will be kept. We will implement a simple score for compliance based on the rate of participation and will use this for sensitivity analyses to check for potential association with effect sizes.

Although the process of IHM is clearly predefined a broad range of individual health behavior actually practiced in the participants’ everyday life has to be anticipated. This variety of options is due to the individually different life situations and thus will not automatically lead to an exclusion from the program or the analysis of the trial. Similarly, participants of the UC group who apparently improve their lifestyle only by written advice need not be excluded.

### Expected benefit

This randomized study will contribute to the evidence of a comprehensive lifestyle program which in the current form has not yet been proved. The program with an active duration of 12 months promises to achieve sustainable effects in weight reduction. Based on a blended learning concept and using web-based e-health tools the program might overcome the well-known experience of many diet programs. Although knowing how lifestyle should be modified it proves to be hard to sustainably make an improved health related behavior reality in everyday life. Furthermore, the multicentre study will show whether comprehensive lifestyle training can be implemented successfully in local health and prophylaxis centres at various spas.

## Additional files

Additional file 1:
**CONSORT 2010 checklist of information to include when reporting a randomised trial*.** (DOC 216 kb) Additional file 2:
**CONSORT 2010 Flow diagram.** (DOC 49 kb)

## Abbreviations

AMG: Arzneimittelgesetz, German pharmaceutical products act
BMI: Body-Mass-Index
BP: Blood pressure
CVD: Cardiovascular disease
DGE: Deutsche Gesellschaft für Ernährung
GCP: Good clinical practice
ICH: International conference on harmonisation of technical requirements for registration of pharmaceuticals for human use
IHM: Individual health management
GP: General practitioner
RCT: Randomized controlled trial
TUM: Technische Universität München
UC: Usual care
