# Supplementary material for: The talent study: a multicentre randomized controlled trial assessing the impact of a ‘tailored lifestyle self-management intervention’ (talent) on weight reduction
Source: BMC Obes. 2015 Oct 1;2:38. doi: 10.1186/s40608-015-0069-x (PMC4589915; doi:10.1186/s40608-015-0069-x)
Supplement: Additional file 2: — CONSORT 2010 Flow diagram. (DOC 49 kb) [file 40608_2015_69_MOESM2_ESM.doc]

**
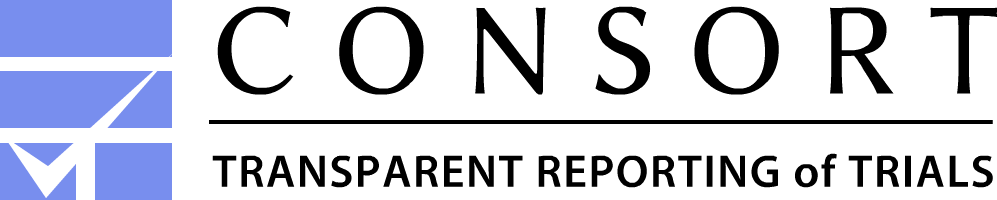
**

**CONSORT 2010 Flow Diagram**

**Allocation**

**Analysis**

**Follow-Up**

**Enrollment**

Assessed for eligibility (estimated n=450)

Excluded

♦  Not meeting inclusion criteria

♦  Declined to participate

♦  Other reasons

Analysed
♦ Excluded from analysis (give reasons) (n= )

Lost to follow-up (give reasons)

Discontinued intervention (give reasons)

Allocated to Individual Health Management intervention (planned n=100)

♦ Received allocated intervention

♦ Did not receive allocated intervention (give reasons)

Lost to follow-up (give reasons)

Discontinued intervention (give reasons)

Allocated to control intervention (planned n=50)

♦ Received allocated intervention

♦ Did not receive allocated intervention (give reasons)

Analysed
♦ Excluded from analysis (give reasons) (n= )

Randomized (planned n=150)

The Talent study: a multicentre randomized controlled trial assessing the impact of a ‘tailored lifestyle self-management intervention’ (talent) on weight reduction

German Clinical Trials Register Freiburg (DRKS)**:** DRKS00006736
